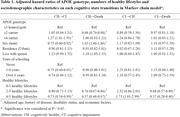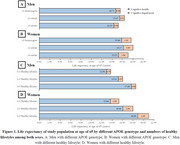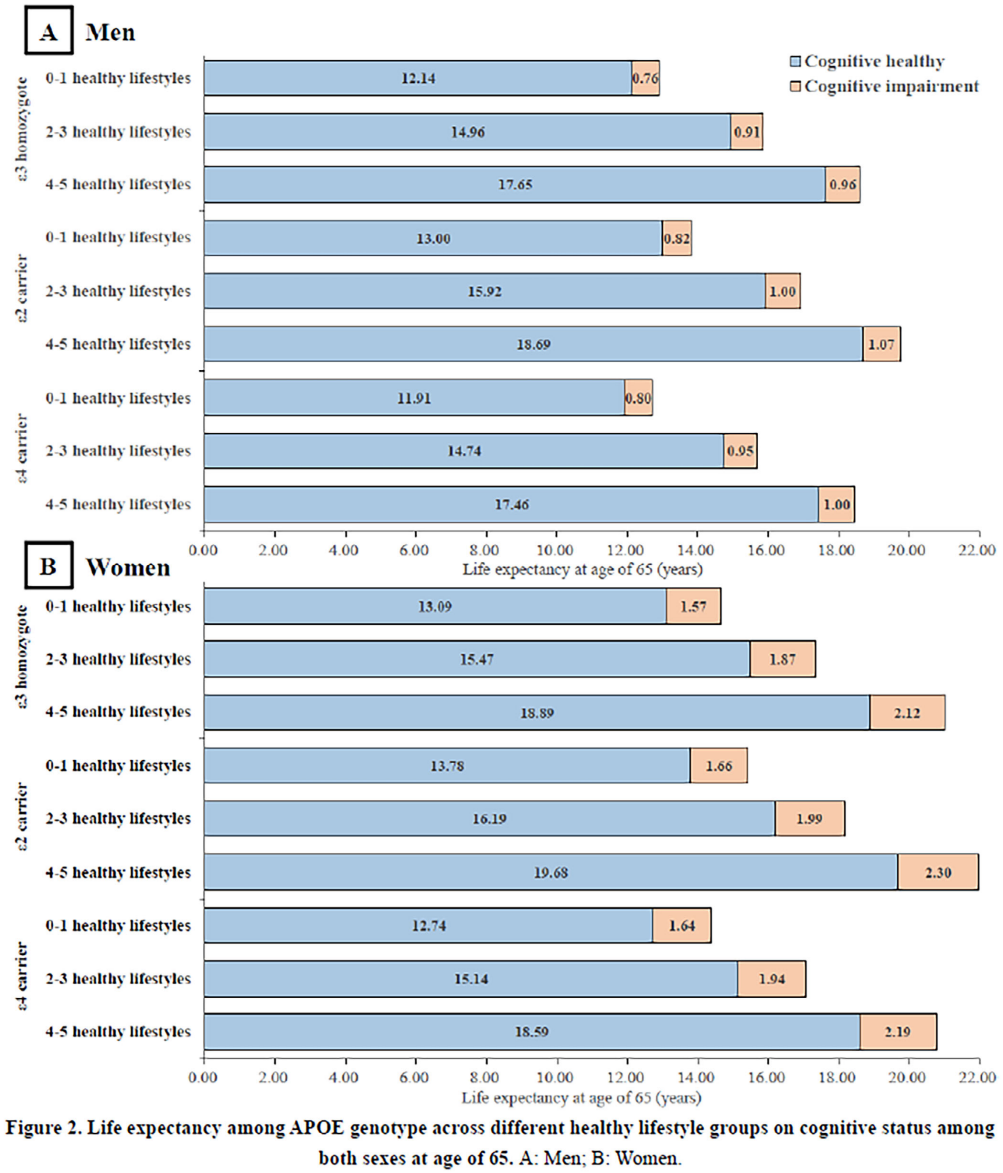# Healthy lifestyle factors outweigh influence of APOE genetic risk on extending cognitively healthy life expectancy

**DOI:** 10.1002/alz70860_101957

**Published:** 2025-12-23

**Authors:** Longbing Ren, Fan Hu, Sebastian Walsh, Xurui Jin, Yang Hu, Shaojie Li, Yuling Jiang, Mingzhi Yu, Yifei Wu, Yi Zeng, Quincy M Samus, Gill Livingston, Yao Yao

**Affiliations:** ^1^ China Centers for Health Development Studies, Peking University, Beijing, Beijing, China; ^2^ Department of Endocrinology, the Second Medical Center & National Clinical Research Center for Geriatric Disease, Chinese PLA General Hospital, Beijing, Beijing, China; ^3^ Cambridge Public Health, University of Cambridge, Cambridge, Cambridgeshire, United Kingdom; ^4^ MindRank AI Ltd., Hangzhou, Zhejiang, China; ^5^ Peking University, Beijing, Beijing, China; ^6^ School of Public Health, China Centers for Health Development Studies, Peking University, Beijing, Beijing, China; ^7^ Duke University, Durham, NC, USA; ^8^ Johns Hopkins University, Baltimore, MD, USA; ^9^ University College London, London, London, United Kingdom

## Abstract

**Background:**

Understanding the interplay between genetic factors and lifestyle choices in cognitive health is crucial for enhancing late‐life quality. This study examines the effects of APOE genotypes and healthy lifestyles on life expectancy with and without cognitive impairment in Chinese older adults.

**Method:**

Utilizing data from the Chinese Longitudinal Healthy Longevity Survey (CLHLS) spanning 15 years. Our cohort comprised 6,488 participants aged 65 and older, whose cognitive function was evaluated using the Mini‐Mental State Examination (MMSE). We assessed five healthy lifestyle factors: no current smoking, no alcohol consumption, diversified diet, being physically active, and maintaining active cognitive engagement. We employed continuous‐time three‐state Markov models to estimate the independent and synergistic impact of APOE genotypes and lifestyle factors on cognitive state transitions and life expectancy.

**Result:**

Compared to APOE ε3 carriers, carriers of the APOE ε4 allele (HR: 1.23, 95% CI: 1.01‐1.59) have a higher risk of transition from cognitively healthy to cognitive impairment, while APOE ε2 carriers (HR: 0.88, 95% CI: 0.78‐0.98) show a decreased risk of transition from cognitively healthy to death (Table 1). Compared to those with 0‐1 healthy lifestyle factor, participants with 4‐5 healthy lifestyle factors substantially extend cognitively health life expectancy (Men: 4.78 years vs. Women: 5.26 years), outweighing such effect of APOE genotypes. Furthermore, benefits on cognitively healthy life expectancy from healthy lifestyle were observed across all APOE genotypes (Figure 2).

**Conclusion:**

These results highlight the potential of public health interventions, i.e., lifestyle modifications, on delaying the onset of cognitive impairment and compressing the lifespan with dementia and cognitive impairment, regardless of genetic predispositions.